# 855. Distinguishing between drug-susceptible and resistant *Neisseria gonorrhoeae* from urine samples by image recognition AI.

**DOI:** 10.1093/ofid/ofad500.900

**Published:** 2023-11-27

**Authors:** Reiichi Ariizumi, Tsubasa Inagaki, Hiroaki Ozaki, Mitsutaka Nakada, Shogo Maeta, Masakazu Nakajima, Makoto Taketani

**Affiliations:** CarbGeM Inc., Shibuya-ku, Tokyo, Japan; CarbGeM Inc., Shibuya-ku, Tokyo, Japan; CarbGeM Inc., Shibuya-ku, Tokyo, Japan; CarbGeM Inc., Shibuya-ku, Tokyo, Japan; CarbGeM Inc., Shibuya-ku, Tokyo, Japan; CarbGeM Inc., Shibuya-ku, Tokyo, Japan; CarbGeM Inc., Shibuya-ku, Tokyo, Japan

## Abstract

**Background:**

Gonorrhea is a major public health concern because it causes complications that can seriously affect maternal and newborn health, including infertility and neonatal eye infections leading to blindness. Antimicrobial resistance (AMR) of the causative pathogen, *Neisseria gonorrhoeae* (*Ng*), emerged shortly after the introduction of antimicrobial agents. This has continued to grow over the past 80 years, culminating in the current outbreak of the gonorrhea superbugs, a broad spectrum of drug-resistant strains of *Ng*. Gonorrhea superbugs can be a major challenge inducing prolonged complications of gonococcal infection and increasing the number of patients with complications.

Here, we demonstrated whether resistant *Ng* can be morphologically distinguished from susceptible *Ng* using our artificial intelligence (AI) assisted image recognition of Gram-stained specimens, which are inexpensive and can be diagnosed in the clinical setting.

**Methods:**

As shown in Figure1, three groups of standard bacterial strains were used for the study; (1) a drug-susceptible *Ng* (ATCC 49226), (2) two drug-resistant *Ng* (NCTC13480 and NCTC13821), and (3) other GNC (ATCC13120: *Neisseria flavescens* and ATCC49143: *Moraxella catarrhalis*). Each bacterial strain was dissolved in female human urine and Gram stained. Image data were collected using a Nikon Eclipse Si microscope at 1000x magnification and iPhone 11.

In total, approximately 600 images were collected, with 60% used for training and 20% for validation. As a final test, 20% of the images were used to test accuracy.

**Figure:**
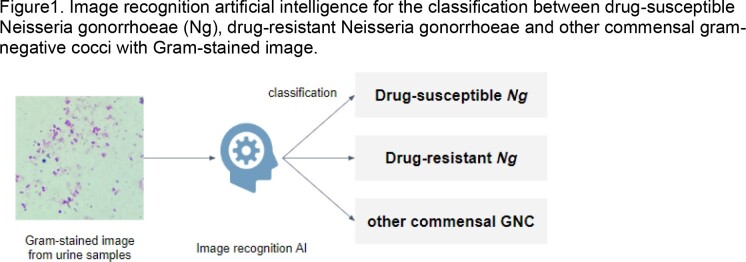

**Results:**

As shown in Table 1, our preliminary AI model achieved 95.3% sensitivity and 96.5% specificity. Since we use Gram-staining, its limit of detection is in theory 1 × 10^5 cells/ml and we confirmed it experimentally.

The classification prediction results.

**Figure:**
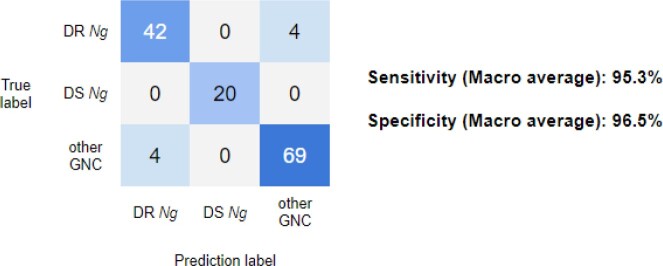

The trained AI was tested on 139 images divided into three groups; (1)Drug-resistant Neisseria gonorrhoeae(DR-Ng), (2)Drug-susceptible Ng (DS-Ng) and (3)other commensal gram-negative cocci (other GNC)

**Conclusion:**

Our preliminary AI model was able to distinguish not only drug-resistant *Ng* from drug-susceptible *Ng*, but also other commensal GNC with Gram-stained specimens, demonstrating the potential for low-cost point-of-care diagnosis of *Ng*. Such a diagnosis will promote the proper use of antimicrobial agents, which can help to prevent the spread of drug-resistant *Ng.*

**Disclosures:**

**Masakazu Nakajima, B. Engineering**, Soiken Holdings: Board Member|Welby Inc.: Board Member|Welby Inc.: Ownership Interest|Welby Inc.: Stocks/Bonds

